# Biological Niches within Human Calcified Aortic Valves: Towards Understanding of the Pathological Biomineralization Process

**DOI:** 10.1155/2015/542687

**Published:** 2015-10-05

**Authors:** Valentina Cottignoli, Michela Relucenti, Giovanna Agrosì, Elena Cavarretta, Giuseppe Familiari, Loris Salvador, Adriana Maras

**Affiliations:** ^1^Department of Earth Sciences, Sapienza University of Rome, Piazzale Aldo Moro 5, 00185 Rome, Italy; ^2^Department of Anatomical, Histological, Legal Medicine and Orthopedics Sciences, Section of Human Anatomy, Electron Microscopy Laboratory “Pietro M. Motta”, Sapienza University of Rome, Via Alfonso Borelli 50, 00161 Rome, Italy; ^3^Department of Earth and Geoambiental Sciences, Aldo Moro University of Bari, Via Orabona 4, 70125 Bari, Italy; ^4^Department of Medical-Surgical Sciences and Biotechnologies, Sapienza University of Rome, Corso della Repubblica 79, 04100 Latina, Italy; ^5^Division of Cardiac Surgery, San Bortolo Hospital, Viale Rodolfi 37, 36100 Vicenza, Italy

## Abstract

Despite recent advances, mineralization site, its microarchitecture, and composition in calcific heart valve remain poorly understood. A multiscale investigation, using scanning electron microscopy (SEM), transmission electron microscopy (TEM), and energy dispersive X-ray spectrometry (EDS), from micrometre up to nanometre, was conducted on human severely calcified aortic and mitral valves, to provide new insights into calcification process. Our aim was to evaluate the spatial relationship existing between bioapatite crystals, their local growing microenvironment, and the presence of a hierarchical architecture. Here we detected the presence of bioapatite crystals in two different mineralization sites that suggest the action of two different growth processes: a pathological crystallization process that occurs in biological niches and is ascribed to a purely physicochemical process and a matrix-mediated mineralized process in which the extracellular matrix acts as the template for a site-directed nanocrystals nucleation. Different shapes of bioapatite crystallization were observed at micrometer scale in each microenvironment but at the nanoscale level crystals appear to be made up by the same subunits.

## 1. Introduction

Calcific aortic valve stenosis (CAVS) is an important public health problem and represents the most common form of valvular heart disease in the industrialized countries [1]. It is strictly associated with the formation of ectopic calcifications within aortic valve leaflets that interfere with cusps opening and lead to ventricular outflow obstruction [2] causing important clinical consequences in terms of mortality and morbidity [3]. To date there is no proven medical therapy to halt CAVS course progression, and surgical or percutaneous valve replacement is the only possible treatment of severe CAVS. The degenerative valve calcification process, however, not only is limited to native heart valves but also affects bioprosthetic implants [4]. Despite much effort devoted to unveil the molecular mechanisms leading to valve calcification, comprehension of the exact process remains uncertain.

Chemically, the calcific deposit within human valve tissue is constituted by a nonstoichiometric apatite, containing high carbonate (CO_3_^2−^) content, from 5% to 10% in weight, and AB-type substitutions in apatite lattice [5, 6], as we previously reported; it is often indicated as “carbonate apatite” or more in general “bioapatite” even if both names are not accepted by the International Mineralogical Association (IMA) Commission on New Minerals, Nomenclature, and Classification (CNMNC) [7]. In this paper, the term “bioapatite” will be used to define the calcium phosphate phase growing in a biological system and forming the ectopic calcification within human heart valve tissue. From a strictly mineralogical point of view such ectopic calcification represents the final product of complex biomineralization processes [8] mediated by biological and physicochemical parameters, as well as the normal mineralized tissues (enamel, dentine, and bone), and falls in the field of calcium phosphate biominerals [9]. Crystal-chemistry features of bioapatite crystals are strongly linked with apatite crystallographic structure [10] and are strictly dependent on the characteristics of the medium in which they form, such as pH, temperature, supersaturation degree, and solution composition [11–13]. More generally a specific microenvironment is required to let biominerals crystallize [8]. This must be a localized zone able to achieve and maintain a sufficient supersaturation degree for crystals nucleation and growth. Therefore the concept of local microenvironment or native biological niche plays an important role in the formation process of biominerals and appears to be the fundamental requirement leading to mineral deposition.

The aim of this paper was to investigate the formation of hierarchical architectures and to determine the spatial relationship existing between crystals and their growth environment. Different experimental techniques, namely, polarized light microscopy, electron microprobe, scanning and transmission electron microscopy, energy dispersive spectrometry, powder X-ray diffraction, Fourier transform infrared spectroscopy, and Raman spectroscopy, were applied on the aortic valve samples to study the calcification process in all its components [5, 6]. Here we discuss the scanning and transmission electron microscopy findings in severely calcified human aortic valves. As biomineral phases are soft materials, subjected to deterioration, amorphization processes, and artifacts formation [14], different preparation methods were used for electron microscopy analyses to monitor the reliability of the experimental results.

## 2. Materials and Methods

### 2.1. Study Subjects

Severely calcified aortic (tricuspid type, *n* = 29; bicuspid type, *n* = 3) and mitral valves (*n* = 4) were obtained from European patients of both sexes (males = 25) and different ages (mean age 72 ± 10, range 41–90 years old). Samples were collected as surgical waste from patients undergoing valve replacement due to severe aortic and mitral valve stenosis without any sign of endocarditis or inflammation. Surgical interventions were performed at the Division of Cardiac Surgery, San Bortolo Hospital, Vicenza, Italy. The institutional committee approved the study and the patients gave written informed consent. Patients' characteristics and clinical data are presented in Table 1; peak and mean gradient were obtained by echocardiographic evaluation [15, 16]. Immediately after surgical excision, part of the heart valve leaflets underwent sample treatment 1; the rest underwent sample treatment 2 to evaluate the same specimen under different methodologies and to reduce hypothetic artifacts due to a single treatment.

### 2.2. Scanning Electron Microscopy (SEM) and Energy Dispersive X-Ray Spectrometry (EDS) Analyses: Sample Treatment 1

Immediately after surgical excision, heart valves were preserved and dehydrated in absolute alcohol. To sterilize the biological material, valve samples were exposed to UV radiation for almost 72 hours at room temperature, which has been proved not to induce any further calcification. A dual beam Zeiss Auriga 405 HR-FESEM with resolution of 1 nm equipped with a Bruker QUANTAX energy dispersive system was used. Investigations were conducted on both uncoated and coated samples. The latter were chromium-coated (5 ÷ 10 nm in thickness) using a Q150T ES turbomolecular-pumped sputtering coater. Low accelerating voltage (<15 kV) was used to obtain information about biomineral/organic structure interface.

### 2.3. Scanning Electron Microscopy (SEM) and Energy Dispersive X-Ray Spectrometry (EDS) Analyses: Sample Treatment 2

**Figure 1:**
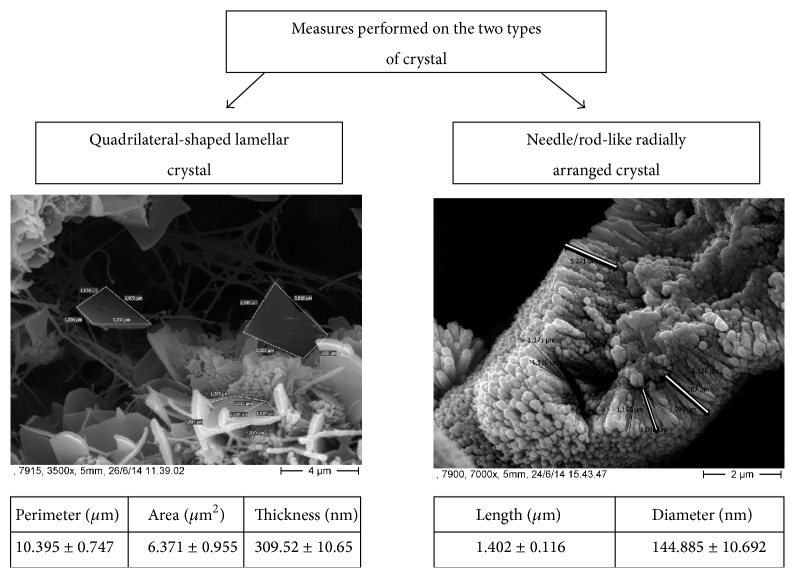
Scanning electron microscope (SEM) analysis on needle/rod-like and quadrangular-shaped crystals and measured considering each type of crystals.

Immediately after surgical excision, heart valves were fixed in glutaraldehyde 2,5% in PBS 0.1 M pH 7,4 immediately after recovery and stored at 4°C for at least 4 days. The parts of the valves in which calcifications had larger dimensions were excised and then postfixed in osmium tetroxide 1% in PBS for 2 hours. Samples were then washed in PBS 0.1 M pH 7,4 and then dehydrated in ascending series of alcohol solutions (30-50-70-95-100%); after this treatment samples were dried in an Emitech K850, “critical point drying” apparatus (Emitech Ltd., Ashford, Kent, England). The dried samples were mounted onto aluminium stubs and then sputter-coated with platinum (~45 nm in thickness) using an Emitech K 550 sputter coater (Emitech Ltd., Ashford, Kent, England). Samples were observed with two different electron microscopes: a Hitachi S400 field emission scanning electron microscope (Hitachi Ltd., Japan) operating at 10 kV and interfaced with a DISS 5 point electronic imaging and analysis system and a ZEISS scanning electron microscope equipped with energy dispersive X-ray spectrometer (SEM-EDS) Zeiss DSM 940A, LEO Elektronenmikroskopie GMbH, Oberkochen, Germany. On needle/rod-like radially arranged crystals, length and diameter were measured. Quadrilateral-shaped lamellar crystal thickness and surface area were measured. Surface area was measured on crystals frontally visible; thickness was measured on crystal laterally placed. Surface area was calculated according to Brahmagupta formula, *s* = √(*p* − *a*)×(*p* − *b*)×(*p* − *c*)×(*p* − *d*), where *p* is the half perimeter and *a*, *b*, *c*, and *d* are the values in micrometers of the four quadrilateral sides (Figure 1). Measures were performed by the digital image processing system DIPS 5 (point electronic GmbH).

### 2.4. Transmission Electron Microscopy (TEM) and EDS Analyses

A JEOL JEM 2010 TEM operating at 200 KV with LaB_6_ source, nominal point resolution of 1.9 Å, and spherical aberration of 0.5 mm was used. An Oxford LINK energy dispersive X-ray spectrometer with a Si (Li) detector and ultra-thin window was used for qualitative chemical analyses. EDS spectra were collected using an acquisition time of 60 s. A single tilt specimen holder was used, and images and diffraction patterns were recorded on Kodak film. The samples were manually grinded in an agate mortar and pestle to a very fine powder. The powdered samples were then subject to an enzymatic attack, with trypsin, in basic pH conditions. After the treatment the powders were exposed to UV radiation for 32–96 hours and then sifted using sieves smaller than 50 *μ*m. This specific treatment allowed us to work with samples with a strongly reduced quantity of collagen. The powder was ultrasonically dispersed in ethanol and subsequently small drops of the slurry were deposited onto a 3 mm diameter Ni-Cu grid coated by a Holey carbon film. The Anticontamination Device (ACD) was used to prevent the contamination of the column due to decomposition of the residual collagen and to minimize the effect of irradiation.

Bright field (BF) images were acquired to define the morphology of the pathological crystals. Selected Area Electron Diffraction (SAED) patterns of large areas, nano-beam electron diffraction (NBD) patterns, and lattice fringes were acquired to determine bioapatite crystal structure. Experimental diffraction patterns after indexing were compared with simulated electron diffraction patterns starting from known crystallographic structures. Simulations were performed with the Java electron microscopy software (JEMS) package (Stadelmann 1999–2008).

### 2.5. Statistical Analysis

Continuous variables are reported as mean ± standard deviation and categorical variables as *n* (%). Computations were performed with SPSS 19 (IBM, Armonk, NY, USA). Comparative analyses could not be performed due to the differences in sample size among groups.

## 3. Results

The electron microscopy images, applied either at the biological specimen or at the inorganic phase, can give clues about the process of growth of pathological bioapatite crystals, from the micrometre up to the nanometre scale, from ex vivo calcified heart valves. The mineralogical features of bioapatite crystals, their spatial relationship with the extracellular matrix, and the formation of hierarchical structures, were determined by the analysis of electron microscopy images as well.

### 3.1. Scanning Electron Microscopy

**Figure 2:**
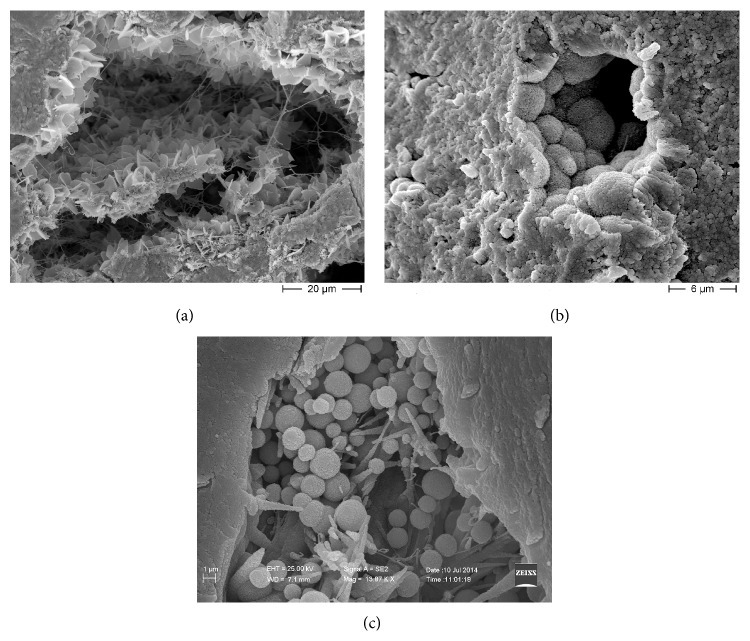
Scanning electron microscopy (SEM) micrographs of niches within human heart valve tissue. (a) Starting from a micrometer scale, within the niches we observed bioapatite deposits in different crystallization shapes. Calcifications appear as lamellar crystals covering the pocket walls. (b) Semispherical deposits containing Ca and P (see EDS spectrum in Figure 6(b)) localized on the walls of the micro-cavity (niches) observed within the organic matrix where Ca and P are below detection limit. Images at higher magnification showed that spherical and semispherical deposits together with lamellar crystals were in turn formed by submicrometer globular structures. (c) Local pocket filled by spherical particles variable in size. Therefore, different crystal shapes observed within the niches appear to be due to different spatial arrangement of submicrometer particles. Scale bars are placed in different ways in the pictures because images were obtained by two different scanning electron microscopes: Figures (a) and (b) are from Hitachi S4000 and (c) is from Zeiss DSM 940.

**Figure 3:**
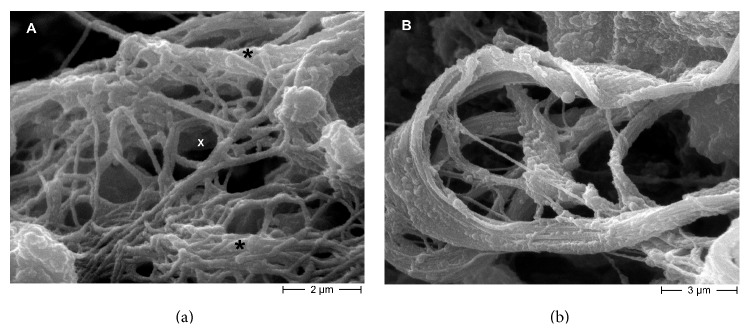
Scanning electron microscopy (SEM) micrographs of extracellular matrix fibers altered architecture. (a) Extracellular matrix fibers are arranged in a loose network (*∗*) or in compact but disorganized bundles (×). Filaments are thicker than normal and show a rough appearance due to the presence of mineral deposits. This is evidence of alteration in normal extracellular matrix deposition process. The three-dimensional mesh creates a microenvironment in which extracellular fluid stagnates, and physicochemical processes of calcium deposition may have taken place. (b) In the foreground, a horseshoe-shaped collagen bundle is visible. In the background wavy, twisted, and bent collagen bundles are present. The space among bundles is crossed by single collagen fibers. This unusual arrangement indicates modification in normal extracellular matrix deposition, and consequently the existence of areas in valve tissue with different stress resistance. Filaments are also thicker due to mineral deposit presence.

Starting from a micrometer-scale and using SEM for biological specimen techniques [14, 17] we highlighted the presence of biological niches within the calcified extracellular matrix, very similar to vugs, small, unfilled cavities inside rock that may be formed through a variety of processes. Within the niches we observed bioapatite deposits in different crystallization shapes (Figure 2). Crystals appear as semispherical deposits covering the pocket walls (Figure 2(a)), as well as lamellar crystals (Figure 2(b)) and spherical particles (Figure 2(c)). The normal architecture of the extracellular matrix fibers is also altered, as shown in Figure 3; fibers are arranged in a loose network or disorganized bundles that can acquire different shapes.

**Figure 4:**
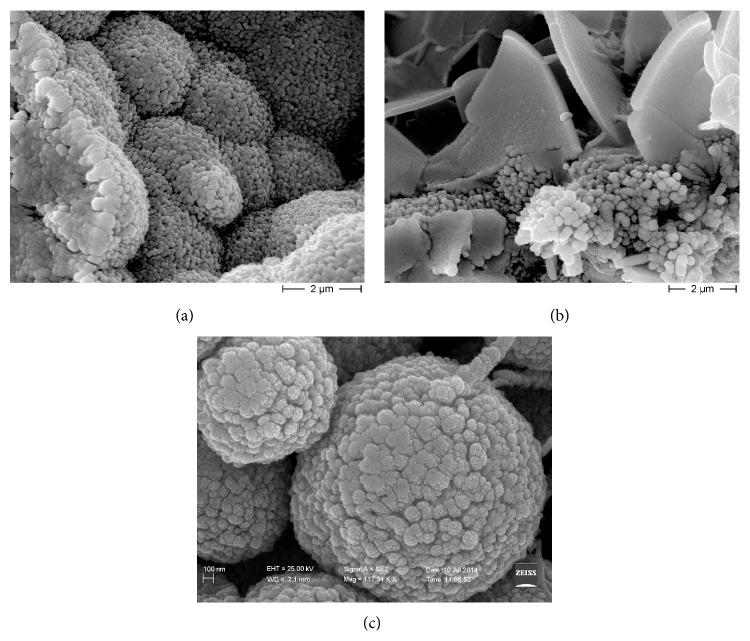
Scanning electron microscopy (SEM) micrographs of submicrometer granular units forming different crystalline morphologies. (a) Higher magnification of semispherical deposits shown in Figure 2(b). Each semispherical deposit is formed by rod-shaped structures with a radial arrangement. Each rod-shaped structure is formed by submicrometer granular units, stacked on each other. (b) Lamellar crystals formed by submicrometer granular units. (c) Higher magnification of micrometric spherical particles shown in Figure 2(c). Smaller units are visible. Scale bar is 100 nm. Scale bars are placed in different ways because images were obtained by two different scanning electron microscopes: (a) and (b) are from Hitachi S4000 and (c) is from Zeiss DSM 940.

Images at higher magnification (Figure 4) showed that semispherical deposits, lamellar crystals, and spherical particles are made up by submicrometer particles such as granular units. Therefore the different crystal shapes observed within the niches appear to be due to different spatial arrangement of submicrometer particles.

Needle/rod-like radially arranged crystals showed a mean length of 1.402 ± 0.116 *μ*m, with a diameter of 145 ± 10.692 nm, while quadrangular-shaped lamellar crystals showed a perimeter of 10.395 ± 0.747 *μ*m, an area of 6.371 ± 0.955 *μ*m^2^, and a thickness of 309.52 ± 10.65 nm (see Figure 1).

### 3.2. High Resolution Field Emission Scanning Electron Microscopy

**Figure 5:**
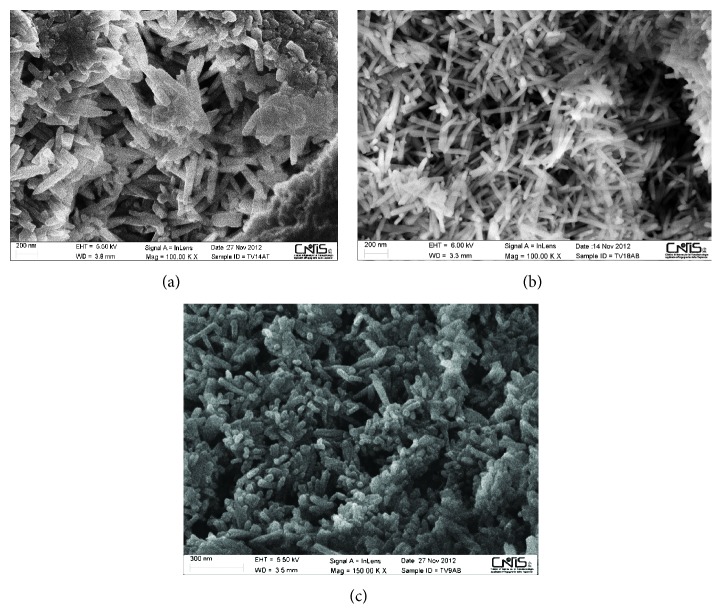
High resolution field emission scanning electron microscopy (FESEM) micrographs of pathological bioapatite nanocrystals. (a) Needle/rod-like nanocrystals distributed as randomly aggregate are visible within a small cavity of the organic tissue of a tricuspid aortic valve. (b) Very thin needle/rod-like nanocrystals grown as randomly aggregate in a small cavity of a bicuspid aortic valve. (c) Needle/rod-like nanocrystals grown onto the organic substrate. Their orientated growth over the organic interface is visible.

Through investigations at nanometer-scale carried out by HR-FESEM, we observed the presence of needle/rod-like bioapatite nanocrystals belonging to different sets of crystals. We observed crystals in the range of 300–680 nm in length and 100 nm in width and smaller ones in the range of 170–200 nm in length and 25–40 nm in width; the first ones appear to be randomly orientated and always localized in small cavities within the organic tissue (Figure 5); despite the similar morphology of these crystals, some differences in shape and size between crystals belonging to different calcified deposits were detected but also within the same mineral deposit have been observed. Otherwise the smaller bioapatite crystals appear to be directly formed onto the organic interface and appear to be oriented with respect to the matrix, indicating a strong interaction between nanocrystals and the organic substrate (Figure 5(c)). High-magnification images also showed the presence of flower-like aggregates of about 300–400 nm in diameter constituted by radiating nanocrystals. At lower magnification degree these aggregate appear as micrometric spheres.

### 3.3. Transmission Electron Microscopy

**Figure 6:**
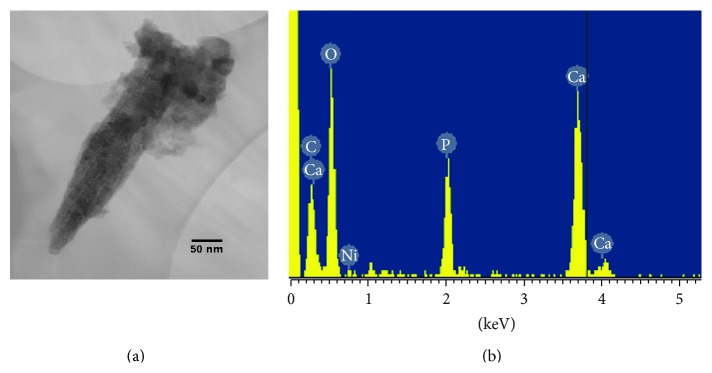
Transmission electron microscopy (TEM) analyses of needle/rod-like pathological crystals. (a) Bright field (BF) image of a needle/rod-like nanocrystal collected from a calcified bicuspid aortic valve. In the bright field (BF) mode of the TEM, an aperture is placed in the back focal plane of the objective lens, which allows only the direct beam to pass. In this case, the image results from a weakening of the direct beam by its interaction with the sample. Therefore, mass thickness and diffraction contrast contribute to image formation: thick areas, in which heavy atoms are enriched, and crystalline areas appear with dark contrast. (b) EDS spectrum corresponding to the nanocrystal showed in panel (a). The spectrum clearly demonstrated the calcium phosphate nature of the nanocrystal. Ni and Cu belong to the 3 mm diameter Ni-Cu grid.

**Figure 7:**
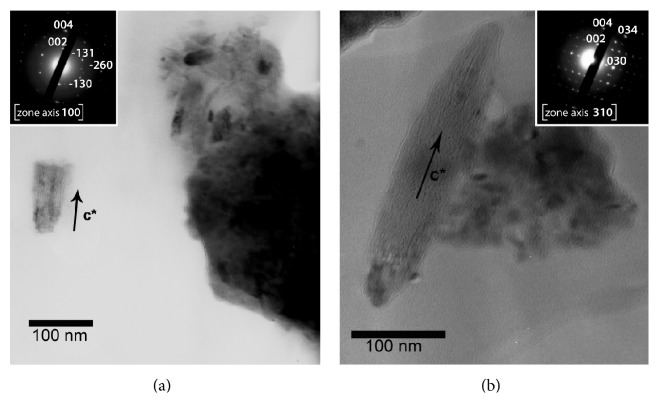
Transmission electron microscopy (TEM) analyses of needle/rod-like pathological crystals. (a, b) Bright field (BF, see Figure 6 for explanation) images and nano-beam electron diffraction (NBD) patterns (in the insets) collected from a calcified bicuspid aortic valve. NBD is a technique that allows determining strain in crystalline materials with a high spatial resolution. A parallel beam with small diameter (~5 nm) is formed in the TEM and scanned across the sample, and diffraction patterns (at a zone axis) are recorded and analyzed for each location. The indices of the diffraction spots indicated in NBD patterns in panels (a) and (b) correspond to interplanar spacings of hydroxylapatite with hexagonal structure (*P6*_*3*_ */m*). The interplanar spacings 002 and 004 are parallel to the elongated form, indicating that nanocrystals elongate parallel to their c-axis.

For a complete characterization, pathological crystals were investigated using TEM images that confirmed the needle/rod-like morphology and the wide range of crystallite size of bioapatite (Figure 6(a)). EDS analyses defined the chemical composition of bioapatite (Figure 6(b)). TEM analyses also confirmed the crystalline character of the pathological phase investigated and allowed us to ascribe it to bioapatite, the hexagonal crystal structure similar to hydroxylapatite (Figure 7).

## 4. Discussion

From our experimental results we hypothesize that the different bioapatite crystallization shapes observed at micrometer- and nanometer-scale are strictly linked to the physicochemical parameters of their native growth niche and to the local condition of the extracellular matrix that represents the framework in which pathological crystals take place. Recent studies have highlighted the essential role of microenvironments in biological systems, indicating the extracellular matrix (ECM) as a local and dynamic niche able to promote the formation of pathological microenvironments [18, 19].

We hypothesize that the spatial organization of collagen fibrils can assume an important role for the delineation of the native growth niches. Studies on bioprosthetic heart valve [20, 21] indicate that calcific deposits are often located within the leaflet tissue, in particular in areas of leaflet higher stress. In these areas it is possible to observe tissue deterioration, distortion in extracellular matrix structure, and small voids as final stage of progressive mechanical tissue damage. These small voids can represent biological niches within the organic matrix, in which subsequently the formation of high-concentrated extracellular fluids takes place, leading to the mineral precipitation and to the activation of the pathological process. Crystals can grow with a regular shape only in a void. In literature, different proposed theories, nonmutually exclusive for vascular calcification, are proposed [22] as loss of inhibition, induction of bone formation, circulating nucleational complexes released from actively remodeling bone, and cell death leading to release of apoptotic bodies and/or necrotic debris.

The differences in shape and size of needle/rod-like nanocrystals observed within the same sample and among different samples might be due to specific conditions of the mineralization niche such as the different degree of supersaturation, the different nucleation frequency, the physicochemical parameters of the starting solution, and the stage of the calcification process. Probably patients' clinical history influences these conditions, but its impact is far from being understood. Taken together, these observations highlight the important role of a purely physicochemical process in the formation of pathological bioapatite crystals within the niches. Indeed these crystals show the typical features of hydroxylapatite crystals precipitated in aqueous solutions [23–26], and their tendency to form radiated aggregates can be the result of a surface energy minimization [27]. The further growth of those elements may follow the Ostwald ripening, with an additional surface energy minimization. However the presence of spherically assembled structures could also be controlled by the local concentration and 3D spatial organization of the fibrillar collagen [28]. Crystals similar to those found in our ex vivo samples were observed in vitro by Tavafoghi Jahromi et al. [29] and Leopold [3]. In particular the similarity of crystals with those observed by Kumon et al. [30] indicates a possible involvement of oxidized lipids in the formation of ectopic calcification within heart valve tissues.

In the matrix-mediated microenvironment, nanocrystals were observed spread through the thickness of the valve tissue and orientated in various ways with respect to the substrate. This can suggest an active role of the ECM in inducing bioapatite nucleation due to its physical properties (rigidity, porosity, insolubility, spatial arrangement, and orientation). The formation of this set of crystals might be also related to a heterogeneous nucleation, probably due to a surface-mineralization process. This mechanism might be mediated by the presence of negatively charged functional groups of the amino acids constituting the proteins of the extracellular matrix [31–33].

The presence of submicrometer units assembled into larger units to form microstructures is typical of biomineralization processes. Biomineralization can develop with a solid phase forming from a solution and then proceeds with the formation of hierarchical structures whose dimensions vary from Ångstroms to millimetres [34–36]. The smallest units aggregate into larger-scaled once producing structures with unusual morphologies. Based on our results, we suggest that the formation of the ectopic biomineralization within the human heart valve follows the same steps (and the same hierarchical organization) of the biomineralization that normally happens in physiologically calcifying tissues [36–38].

The spherical particles (matrix vesicles), similar to those reported by Bertazzo et al. [39], were always observed in strict association with organic filaments. Energy dispersive spectroscopy (EDS) analyses acquired on these particles confirmed that they are made up of calcium phosphate, sulphur (S), and azote (N). This means that in the vesicles there are also proteins that act on the growth of these mineralized phases [40], morphologically different from bioapatite nanocrystals. This is confirmed from previous studies on the involvement of specific proteins (glycosaminoglycans, GAG) and lipoproteins in controlling the size and shape of hydroxylapatite [41–43].

Our nanoscale observations indicate that the formation of pathological bioapatite nanocrystals within heart valves is related to the presence of a highly heterogeneous mineral deposit. Different growth processes may occur in different microenvironments (each one with its own physicochemical characteristics) influencing the shape of the biomineralization. The presence of compartmental niches within the extracellular matrix assumes a relevant role in the formation of ectopic biomineralization in human heart valves as well as the action of the organic substrate on the crystals features.

We suppose bioapatite nanocrystals to be the first mineralized product within the organic framework and we consider their appearance as the pivotal step in disease initiation and progression in agreement with the study of Ewence et al. [44] that have demonstrated the bioactivity of calcium phosphate crystals as function of their crystal size. Bioapatite nanocrystals in the soft tissues of the human heart valve may activate an inflammatory process that increases and strengthens, within the endocardial valve layer, leading to irreversible pathological conditions such as valve structure disorganization, excessive GAG accumulation, elastic fiber fragmentation, cell activation, and cell death. This pathological condition once activated may aggravate the inflammatory response started by inorganically formed bioapatite nanocrystals and lead to a severe calcification process. In addition, autophagy, a critical mechanism for the aging process, involved in the regulation of cardiac homeostasis and response to stress, is deeply implicated in this process, as hypoxia, nutrient deprivation, and ischemia are a strong stimulus for autophagy [45]. If autophagia becomes upregulated, the digestion of damaged proteins and organelles can create the perfect conditions for the niche, as a newly created cavity, resulting from cell death that can host calcification process.

### 4.1. Limitations

We are aware that several limitations exist, but to our knowledge this is the first report to describe the two different growth processes linked to the different microenvironments. Due to the small sample size and the prevalence of tricuspid aortic valves, we could not compare results of the three different types of valves, to highlight significant differences in their biomineralization. As we already reported a significant difference in the content of calcium and phosphorous [6] between tricuspid and bicuspid aortic valves, we are convinced that architectural differences in collagen fibers and nanocrystal orientation exist and will be the object of a future study. A larger sample size would allow us also to correlate clinical data to the different forms of biomineralization and to better investigate the multifactorial pathogenesis of this complex disease and this study is actually ongoing. This is not an in vivo study to investigate biominerals crystal growth in a follow-up over time, but in its nature of ex vivo observational study we are convinced that different stages of growing are represented in a whole heart valve, as we analyzed heavy calcified samples but also areas with no appearance of visible macrocalcification that were indeed present as microcalcification at a submicrometer scale.

## 5. Conclusions

Our findings on pathological bioapatite nanocrystals growth processes may be helpful for understanding the biomineralization that affects human heart valve tissues and we stress on the importance that the mineralogical approach joined to the medical and biological fields can have to resolve this multidisciplinary phenomenon. To understand the formation of biomineralized deposits in the human heart tissues it is important to focus the attention on the nucleation of the bioapatite crystals, because all crystalline material, including bioapatite, can grow only after a nucleus is formed. In particular, biominerals growth in preferential sites for nucleation within complex crystal composites and the generation of crystallites with specific crystallographic orientations can be explained with a potential active control during the nucleation stage, operated by lipoproteins or other molecules, still not extensively investigated yet.

Therefore mineralogical and biological electron microscopy analyses on pathological bioapatite crystals may be extremely important to gain information about their crystallization pathway and on factors involved in the heart valve calcification process.

## Figures and Tables

**Table 1:** Patients characteristics.

Characteristic	Overall *N* = 36	Tricuspid aortic valve *N* = 29	Bicuspid aortic valve *N* = 3	Mitral valve *N* = 4
Age, y	72.4 ± 10	74.5 ± 7.8	55 ± 15	69 ± 9
Males, *n*	25 (69.4%)	20 (69%)	3 (100%)	2 (50%)
BMI, Kg/cm^2^	25.1 ± 5.5	25.5 ± 6	22.2 ± 3.7	22.4 ± 2
BSA, m^2^	1.8 ± 0.2	1.8 ± 0.2	1.7 ± 0.1	1.7 ± 0.2
Peak gradient, mmHg	71.2 ± 20.5	70.5 ± 23	88.4 ± 18	9 ± 3.5
Mean gradient, mmHg	55.8 ± 16.2	53.4 ± 18.3	58.8 ± 14	6 ± 3.4
Associated CAD	13 (36%)	13 (44.8%)	0 (0%)	0 (0%)
Dialysis	0 (0%)	0 (%)	0 (0%)	0 (0%)
Osteoporosis	22 (61%)	21 (72%)	0 (0%)	1 (25%)

BMI: body mass index; BSA: body surface area; CAD: coronary artery disease.
